# Mathematical Creativity in Adults: Its Measurement and Its Relation to Intelligence, Mathematical Competence and General Creativity

**DOI:** 10.3390/jintelligence9010010

**Published:** 2021-02-17

**Authors:** Michaela A. Meier, Julia A. Burgstaller, Mathias Benedek, Stephan E. Vogel, Roland H. Grabner

**Affiliations:** 1 Educational Neuroscience, University of Graz, Universitätsplatz 2, 8010 Graz, Austria; burgstaller.julia.priv@gmail.com (J.A.B.); stephan.vogel@uni-graz.at (S.E.V.); roland.grabner@uni-graz.at (R.H.G.); 2 Creative Cognition Lab, University of Graz, Universitätsplatz 2, 8010 Graz, Austria; mathias.benedek@uni-graz.at

**Keywords:** mathematical creativity, intelligence, mathematical competence, general creativity

## Abstract

Mathematical creativity is perceived as an increasingly important aspect of everyday life and, consequently, research has increased over the past decade. However, mathematical creativity has mainly been investigated in children and adolescents so far. Therefore, the first goal of the current study was to develop a mathematical creativity measure for adults (MathCrea) and to evaluate its reliability and construct validity in a sample of 100 adults. The second goal was to investigate how mathematical creativity is related to intelligence, mathematical competence, and general creativity. The MathCrea showed good reliability, and confirmatory factor analysis confirmed that the data fitted the assumed theoretical model, in which fluency, flexibility, and originality constitute first order factors and mathematical creativity a second order factor. Even though intelligence, mathematical competence, and general creativity were positively related to mathematical creativity, only numerical intelligence and general creativity predicted unique variance of mathematical creativity. Additional analyses separating quantitative and qualitative aspects of mathematical creativity revealed differential relationships to intelligence components and general creativity. This exploratory study provides first evidence that intelligence and general creativity are important predictors for mathematical creativity in adults, whereas mathematical competence seems to be not as important for mathematical creativity in adults as in children.

## 1. Introduction

In our rapidly changing world where technological and scientific advancements affect our everyday life, creativity is an increasingly important aspect (Sternberg and Lubart 1999; Leikin and Pitta-Pantazi 2013). Creativity does not only help us to adapt to changes, but is also a prerequisite for technical and scientific advances (Leikin 2013). Research on creativity in the domain of mathematics has recently moved in the focus of attention (Assmus and Fritzlar 2018; Sak et al. 2017). On the one hand, mathematics, as a science, serves as the foundation for various other scientific and technological discoveries. On the other hand, creativity in mathematics is necessary to ensure the growth of the field itself, and it is essential for solving mathematical problems, for which there is no learned solution. This applies regardless of whether a mathematician tries to solve a complex open problem or a primary school student tries to solve an unfamiliar problem (Leikin and Pitta-Pantazi 2013). Despite its importance and the increasing research over the past years, much about the concept of mathematical creativity is still unknown. One of the main research gaps, which this study tries to fill, is that there is no empirically validated mathematical creativity measure for adults, because mathematical creativity has mainly been investigated in children and adolescents so far (i.e., Leikin 2013; Schoevers et al. 2018). The few studies that measured mathematical creativity in an adult sample only used one or two items, and mathematical creativity was not the main focus of the research (Baer 1993; Chen et al. 2006). Consequently, it is unknown which cognitive factors uniquely predict mathematical creativity in adults, and if abilities that were found important for mathematical creativity in children (i.e., intelligence, mathematical competence, and general creativity) are equally relevant for adults.

### 1.1. Definition and Measurement of Mathematical Creativity

Similar to general creativity, the concept of mathematical creativity has been defined in various ways. The most common definition, which will also be used in this study, is that “mathematical creativity is the process that results in unusual or novel as well as useful solutions to a given mathematical problem” (Sriraman 2005). This is commonly assessed by mathematical divergent thinking tasks. In mathematical divergent thinking tasks, participants have to find as many creative solutions as possible to a particular mathematical problem. In the majority of studies, mathematical creativity is measured by more than one divergent thinking task to cover different fields of mathematics. Most common are tasks drawing on numerical, arithmetic aspects (e.g., “Provide as many different mathematical operations using the numbers 3, 4, 20, 24, 72 and the operation symbols +, −, ×, /, =”, Karwowski et al. 2020) as well as tasks drawing on figural, geometric aspects (e.g., “Divide a square with a side length of 4 cm for any number of equal parts”, Karwowski et al. 2020). The solutions to these problems are then scored in terms of fluency, flexibility, and originality. Fluency is the number of mathematically correct answers that are given, flexibility is the number of different categories all answers of one individual fall into, and originality is how unusual or novel an answer is compared to a reference group (Schoevers et al. 2018).

### 1.2. Intelligence and (Mathematical) Creativity

Creativity is not an isolated ability but rather requires distinct but interrelated resources. Among others, two of these resources are intellectual abilities and knowledge (Sternberg and Lubart 1991; Simonton 2014). First and foremost, the role of intelligence for creativity has been subject to long-standing research and debate (Kattou and Christou 2017; Kaufman and Plucker 2018). While some, predominantly older, studies found no or only small relationships between intelligence and creativity and therefore consider them to be unrelated (e.g., Kim 2005), more recent studies found quite substantial relationships. Especially when scoring the creative quality of responses (subjective judgement of originality by raters) and using latent variable models (Silvia 2015; Benedek et al. 2014).

A similar picture emerges for the relationship between intelligence and mathematical creativity. Two cross-sectional studies with fourth graders showed that intelligence is positively correlated with mathematical creativity. While the studies used conceptually different tests to assess intelligence, the results showed a consistent positive association with creativity. Kroesbergen and Schoevers (2017) used a Dutch intelligence test for education level (Van Dijk and Tellegen 2004), which measures verbal and spatial reasoning, and found a significant relationship with mathematical creativity of *r* = 0.53. Schoevers et al. (2018) used the Raven’s Standard Progressive Matrices (Raven 1998), which are used to assess fluid intelligence, and also found a significant association with mathematical creativity of *r* = 0.55. A more nuanced picture of this association was provided in two other studies. Kahveci and Akgul (2019) showed that gifted individuals, operationalized as having an IQ above 130, outperformed individuals with an IQ below 130 in a mathematical creativity task. Kattou and Christou (2017) found that fluid intelligence, as measured with the Naglieri Nonverbal Ability Test (Naglieri 1997), is a significant predictor of the latent factor of mathematical creativity (*r* = 0.50). In addition, Kattou and Christou (2017) investigated whether the correlation between intelligence and mathematical creativity is comparable in three groups of students differing in their intelligence scores (lowest 5%, average, highest 5%). While there was a moderate positive correlation between intelligence and mathematical creativity in the average group, no significant correlations were found in the lowest and highest 5% groups. The authors interpret these results in the context of the threshold theory (e.g., Karwowski et al. 2016), which argues that intelligence is a necessary but not sufficient precondition for creativity. However, the sample sizes of the lowest 5% (*N* = 18) and of the highest 5% (*N* = 29) group were quite small, especially in comparison to the average group (*N* = 429). Therefore, these findings have to be interpreted cautiously. Although a positive association between intelligence and mathematical creativity has been found in children, we do not know if this association might differ in adults.

One additional potential shortcoming of all these studies mentioned above, which assessed the relationship between intelligence and mathematical creativity, is that only general intelligence or fluid intelligence were considered. However, creativity research suggests that different components of intelligence can be relevant in different creative domains (Kellner and Benedek 2017; Beaty and Silvia 2013; Forthmann et al. 2019; Cho et al. 2010). Therefore, not only general intelligence but also sub-facets of intelligence (i.e., numerical, figural, and verbal intelligence) should be considered for the prediction of mathematical creativity, which has been done in this study.

In addition to intelligence, mathematical competence should also be taken into account as a predictor for mathematical creativity. However, intelligence and mathematical competence are not only related to mathematical creativity, but also related to each other. Intelligence is one of the most important predictors for educational achievement and especially important for mathematical achievement (Roth et al. 2015). For example, intelligence, assessed at the age of 11, accounts for nearly sixty percent of the variance within mathematical competence assessed at the age of 16 (Deary et al. 2007). Hence, the role of intelligence and its sub-facets needs to be kept in mind when assessing the relationship between mathematical competence and mathematical creativity, which is discussed in the next section.

### 1.3. Mathematical Competence and Mathematical Creativity

In order to be creative, at least some familiarity with the topic as well as some knowledge within the domain is required (e.g., Csikszentmihalyi 1996; Haylock 1997; Karwowski et al. 2016). In addition, having a higher expertise in a domain makes creativity in this specific domain more likely (Baer 2015). This is the reason why mathematical competence, mathematical knowledge, mathematical abilities, or mathematical achievement are assessed together with mathematical creativity in the majority of studies. Previous studies have reported a positive relation between mathematical knowledge, measured with mathematical ability tests, and mathematical creativity in children and adolescents (e.g., Haylock 1997; Kattou et al. 2013; Tubb et al. 2020). Correlational analyses showed small (*r* = 0.20, Stolte et al. 2020) to large (*r* = 0.61, Kattou et al. 2013) effects in children aged 7 to 12.

As one of the few studies on mathematical creativity in adults, Chen et al. (2006) assessed mathematical creativity in 158 undergraduate students with two adapted tasks from Haylock (1987), who originally constructed these tasks for school children. Participants were asked to generate multiple responses, which varied greatly in originality. The first task was called Cutting Rectangles Task, in which four rectangles were presented. Two of these rectangles had to be separated into four component rectangles by drawing straight lines, and two had to be separated into nine component rectangles. In the second, task Nine-Dot Areas Task, participants were given three four cm^2^ squares (represented by nine points with a respective distance of one cm) and had to form shapes with a size of two cm^2^ within by connecting the points with drawn lines. Items were scored by trained undergraduate research assistants with objective coding schemes. For example, in the Cutting Rectangles Task, products were coded as low creative if the lines were only vertical or only horizontal leading to same sized rectangles. Products were coded as medium creative if there were vertical and horizontal lines of different lengths leading to rectangles of varying size. Products were coded as highly creative if they featured more complex configurations leading to three-dimensional or imbedded rectangles. Mathematical creativity showed a medium correlation (*r* = 0.35) with the quantitative scores of the SAT. However, as SAT scores are strongly predicted by general intelligence (Hannon 2016), and this study did not include a measurement of intelligence, it is hard to make assumptions about the unique relationship between mathematical creativity and mathematical competence in adults.

Importantly, correlational analyses do not provide information about the direction of the relationship between mathematical creativity and mathematical competence. As knowledge within a domain is seen as a necessity for creative performance, Schoevers et al. (2018) used structural equation modeling (SEM) to show that a model in which mathematical competence predicted mathematical creativity fitted the data better than models in which mathematical creativity predicted mathematical competence. A similar view was put forward by Haylock (1997), who suggested that mathematical abilities limit, but do not determine mathematical creativity. In other words, pupils who show low mathematical abilities do not have sufficient mathematical knowledge and competence to produce highly creative mathematical solutions. However, there are still individuals who show high mathematical competence but perform poorly at creative mathematical thinking. Karwowski et al. (2020) further examined the assumed necessary-but-not-sufficient link between mathematical competence and mathematical creativity in a large sample of sixth-grade elementary school students and first grade middle school students (*N* = 2372) using NCA. The results of the analysis demonstrated a significant moderate-to-strong effect size, which indicated that high scores in mathematical creativity were hardly possible when the scores in mathematical competence were low. Extending this evidence, the authors applied a quantile regression analysis, which models the relationship between the variables at different points of the distribution of the dependent variable. The results of this analysis showed that mathematical competence was equally important for explaining low, medium, and high mathematical creativity.

Other studies have explored the relationship between mathematical creativity and mathematical competence in more detail. Kattou et al. (2013), for example, tested whether mathematical abilities are a component of mathematical creativity or vice versa by comparing different models using confirmatory factor analysis (CFA). They found somewhat stronger support for a model in which mathematical creativity is a subcomponent of mathematical abilities, and the authors argued that this result is in accordance with the view that mathematical creativity predicts mathematical abilities. This finding is in line with other results indicating that mathematical creativity might be a prerequisite for the development of mathematical competence (Mann 2005; Leikin 2007). Mathematical creativity was shown to explain approximately 50% of the variance in mathematical competence (Bahar and Maker 2011). Consequently, Tyagi (2017) argued that the relationship between mathematical competence and mathematical creativity might be a bidirectional rather than an unidirectional relationship.

To sum up, there is strong evidence for a positive relationship between mathematical competence and mathematical creativity. Although evidence for both directions of this relationship exists, this study focused on the predictive value of mathematical competence for mathematical creativity, in line with the theory that knowledge is necessary for being creative.

### 1.4. General Creativity and Mathematical Creativity

Another recurring research question is whether intelligence and mathematical proficiency are sufficient to explain mathematical creativity or if general creativity has additional predictive value. This concerns the question whether mathematical creativity is a manifestation of general creativity or a largely independent capacity. More generally, it addresses to some extent the long-standing question of whether creativity is a domain-specific phenomenon (i.e., different specific creative capacities are required in different creativity domains) or a domain-general phenomenon (i.e., contributing to creative competences in various domains; e.g., Baer 2010; Plucker and Zabelina 2009).

Baer (1993) asked eighth-grade students to create a poem, a story, a mathematical word problem, and an interesting mathematical equation, which were afterwards rated on creativity by experts. After the variance attributed to math and verbal abilities was controlled for, neither the creativity in the production of a word problem nor of an equation significantly correlated with creativity in poetry and story writing. Kattou et al. (2015) measured mathematical creativity with the Mathematical Creativity Test (MCT; Kattou et al. 2013) and general creativity with items (one verbal and one figural) from the Torrance Test of Creative Thinking (TTCT; Torrance 1966). The findings from this work suggested a dissociation: students who were identified as creative with one instrument were not necessarily identified as creative in the other instrument. A CFA confirmed the appropriateness of the domain-specific model of creativity by comparing the two alternative theoretical models. Likewise, Huang et al. (2017) showed that even though mathematical creativity (measured with the Nine-Dot Areas Task) is modestly positively correlated with general creativity (measured with one verbal and one figural item), only mathematical competence, but not general creativity, can effectively explain the variance within mathematical creativity. This is in line with other studies who provided evidence that the association between general creativity and mathematical creativity is significantly smaller than the association between mathematical competence and mathematical creativity (e.g., *R*^2^ = 3% for general creativity vs. *R*^2^ = 10% for mathematical competence, Jeon et al. 2011; *r* = 0.19 for general creativity vs. *r* = 0.42 for mathematical competence, Lin and Cho 2011). Those studies indicate that creativity is rather domain-specific.

In contrast to these findings, there are also studies that have provided evidence that general creativity is a significant predictor of mathematical creativity, even when mathematical competence is taken into account (Livne and Milgram 2006). Further, there is evidence that general creativity and mathematical competence are almost equally good predictors for mathematical creativity (e.g., *r* = 0.40 for general creativity vs. *r* = 0.34 for mathematical competence, Schoevers et al. 2018; *r* = 0.20 for general creativity vs. *r* = 0.20 for mathematical competence, Stolte et al. 2020). This rather speaks for a domain-general view of creativity.

Apart from Baer (1993) all of the studies mentioned in this section used divergent thinking tasks to assess general creativity as well as divergent thinking tasks in the mathematical domain to assess mathematical creativity. Therefore, the association between general and mathematical creativity may differ if creativity would be assessed differently. Concluding from the presented results above, there is evidence for both a domain-general and a domain-specific account, which leads to the assumption that creativity may be partly domain-general and domain-specific (Schoevers et al. 2018; for a relevant theoretical model, see Baer and Kaufman 2005). However, the exact nature of the relationship between mathematical creativity and general creativity still has to be resolved, and the nature of this association might differ in adults.

### 1.5. The Present Study

The current study investigated the question of how different cognitive abilities (i.e., intelligence, mathematical competence, and general creativity) are related to, and uniquely predict, mathematical creativity in adults. To achieve this goal, we developed a mathematical creativity measure for adults (MathCrea). Following the theoretical concepts of assessing mathematical creativity with divergent thinking tasks, we adapted available tasks (covering numerical as well as figural aspects of mathematical creativity) used in children for adults. Consequently, the first part of this study focused on the evaluation of the psychometric properties and structure of the MathCrea. The second part of this study focused on the question of how intelligence, mathematical competence, and general creativity are related to mathematical creativity in adults and what unique predictive value they have. In line with the reviewed literature focusing mostly on children and adolescents, we expected to find a positive relationship between intelligence and mathematical creativity (e.g., Kroesbergen and Schoevers 2017; Kahveci and Akgul 2019; Kattou and Christou 2017), and differentiated relationships between the sub-facets of verbal, numerical, and figural intelligence and mathematical creativity. Further, we expected a positive relationship between mathematical competence and mathematical creativity (e.g., Chen et al. 2006; Bahar and Maker 2011; Haavold 2016; Karwowski et al. 2020; Schoevers et al. 2018). Additionally, based on the discussed results (e.g., Lin and Cho 2011; Schoevers et al. 2018), we expected general creativity to have a positive relationship with mathematical creativity in adults.

## 2. Materials and Methods

### 2.1. Participants

We collected data from subjects with a wide range of educational backgrounds and professions. That allowed the examination of mathematical creativity in the broadest possible context. Participants were recruited by sending out an email to all students of the local university, and the investigators approached friends and acquaintances. Overall, 103 adults participated. All participants were healthy and did not have a learning disorder (dyslexia or dyscalculia). Three participants were excluded from data analysis, two individuals because of a large amount of missing data in the main mathematical creativity measure*,* and one individual due to insufficient language proficiency. The final sample consisted of 100 adults between 18 and 37 years (*M* = 23.38, *SD* = 3.45; 53 females). In this sample, 74 were university students, 52% of whom were studying psychology, whereas the others exhibited diverse university majors (from mathematics, physics, law, and linguistics to pedagogy). The other 26 individuals were fully employed, 23 of whom had a university degree, while three individuals completed only secondary education. All participants gave informed consent, and the study was approved by the local ethics committee.

### 2.2. Materials

#### 2.2.1. Mathematical Creativity Measure for Adults (MathCrea)

Mathematical creativity was measured with four tasks that were adapted from tasks used before to measure mathematical creativity in children. Two of the tasks used numerical material and the other two used figural material. Within each domain, one task focused on the generation of mathematical products and the other on the identification of similarities. Participants were instructed to produce as many solutions as possible for each problem within three minutes.

The first numerical task (Numerical Generate) was originally used by Prouse (1964). The original task was to use the numbers (2, 3, 8) and the symbols +, −, (), ×, /, to create as many correct equations as possible. In the modified task used in the present study, the task was slightly adjusted to better suit adult subjects by implementing additional rules. Specifically, the same number could be used a maximum of two times in succession, and only the numbers (2, 3, 8) but no operation symbols were allowed in the solution. The additional rules were implemented with the intention to increase the difficulty of the task. Some examples solutions given by participants were “3 – 3 + 2 = 2”, “(2 + 8)/2 – 3 = 2”, and “((8/3 − ((3 + 2)/3)) × 2 = 2”.

The second numerical task (Numerical Similarities) is adapted from Haylock (1987). In the original task, participants were asked to form subsets of numbers ranging from 2 to 16 by identifying similarities between these numbers. For example, the numbers “2, 3, 5, 7, 11, 13” can be summarized into the subset “prime numbers”. In the present study, the task was adjusted towards higher task complexity by focusing on two specific numbers, which substantially reduces the solution space. Specifically, the participants had to identify as many similarities as possible between the numbers 16 and 36 and to write them down. Some example solutions given by participants were “both include the number 6”, “both numbers are composed of an even number and an odd number”, and “the digit sum of both numbers is larger than 6”.

**Figure 1:**
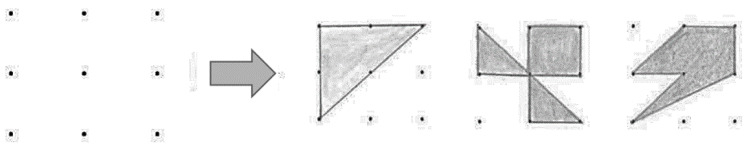
Visualization of the item Figural Generate on the **left**. Three example solutions given by participants are presented on the **right**.

The first figural task (Figural Generate) was the “nine-dot-areas” problem by Haylock (1987). Subjects had to form shapes with a size of exactly two cm² within given four cm² squares (represented by nine points with a respective distance of one cm) by connecting the points with drawn lines. The item and some examples of possible solutions in the figural tasks are presented in Figure 1.

**Figure 2:**
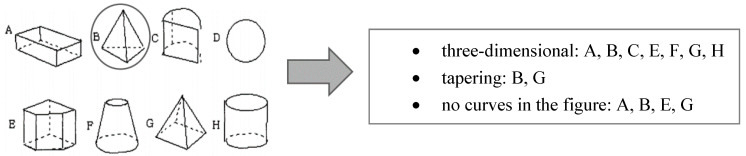
Visualization of the item Figural Similarities on the **left**. Three example solutions given by participants are presented on the **right**.

In the second figural task (Figural Similarities) by Becker and Shimada (1997), eight different geometric figures (figure A–figure H) were shown, in which common properties between figure B and one or more of the other figures had to be found. The task and some examples of possible solutions are presented in Figure 2.

The responses to each task were scored for fluency, flexibility, and originality. This scoring was consistent with the scoring of well-established mathematical creativity tests used in children and adolescents (e.g., MCT; Kattou et al. 2013; MST; Leikin 2013). Fluency was operationalized as the number of correct answers. Flexibility was operationalized as the number of categorically different responses. Examples for the different categories and for answers within each category can be found in Table A1. Originality was operationalized in two different ways. First, we computed an infrequency score reflecting the uniqueness of a response in our sample. This scoring was done according to the guidelines of the MCT from Kattou et al. (2013). An answer got a score of five points if this answer was given by less than 1% of the sample, four points if the answer was given by 1–5%, three points if the answer was given by 6–10%, two points if the answer was given by 11–20%, and one point if the answer was given by more than 20% of the sample. As the infrequency scoring is known to reflect just the unusualness of responses, we additionally obtained subjective creativity ratings that capture the creative quality of responses in a more holistic way (e.g., cleverness, elegance, etc.; Reiter-Palmon et al. 2019; Silvia et al. 2008). Five independent raters scored all answers from 1 (not creative) to 5 (very creative). The inter-rater reliability was evaluated through the intraclass correlation coefficient (ICC). ICCs showed that all four tasks had excellent inter-rater-reliability calculated at response level (Numerical Generate: ICC = 0.91; Numerical Similarities: ICC = 0.91; Figural Generate: ICC = 0.92; Figural Similarities: ICC = 0.89; Koo and Li 2016).

**Figure A1:**
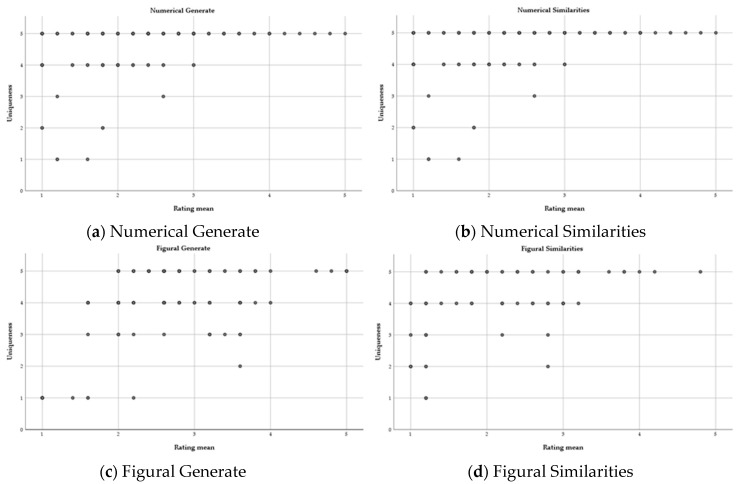
Correlation between originality assessed by infrequency and originality assessed by ratings (mean score of five independent raters) for all four tasks: (**a**) Numerical Generate; (**b**) Numerical Similarities; (**c**) Figural Generate; (**d**) Figural Similarities.

The correlation between the originality scoring based on infrequency and on ratings at response level was moderately high (Numerical Generate: *r*(98) = 0.35, *p* < 0.001; Numerical Similarities: *r*(98) = 0.29, *p* = 0.002; Figural Generate: *r*(98) = 0.41, *p* < 0.001; Figural Similarities: *r*(98) = 0.47, *p* < 0.001). A closer look at the scatter plots (depicted in Figure A1) offered further insight on the specific nature of the relationship between infrequency and ratings. Answers that were rated as very creative were hardly possible when the infrequency of the answer was low. In contrast, an answer being very unusual did not indicate that the answer necessarily was rated as very creative. This indicated that infrequency is necessary but not sufficient for high creativity ratings (comparable to the relationship between mathematical competence and mathematical creativity). Assuming that infrequency and ratings both measured relevant aspects of response originality (Silvia et al. 2008), we integrated both scores into a combined mean score for originality. For fluency, flexibility, and originality, summative scores were used accordingly to studies using the MCT (e.g., Kattou et al. 2013; Schoevers et al. 2018; Stolte et al. 2018). Those summative scores were than z-standardized for all further analyses. For the separate analyses of quantitative and qualitative aspects of mathematical creativity, originality was averaged by fluency before z-standardization to obtain originality scores that are not biased by fluency confounds (Benedek et al. 2013; Silvia et al. 2008) see Section 3.3.

#### 2.2.2. Intelligence

Intelligence was assessed using the well-established Berlin Intelligence Structure Test (BIS-T), which was developed based on the Berlin Intelligence Structure Model (Jäger et al. 1997). The model assumes intelligence as an ability consisting of two parts (general intelligence and content-related abilities). In the present study, the short form of the BIS was used to measure intelligence. It included 15 tasks and covered all three domains of intelligence (numerical, verbal, and figural) as well as the four operational abilities (processing speed, memory, reasoning, and creativity) described in the theoretical model. The internal consistency of the separate scales is good (Cronbach’s α = 0.75–0.89). The processing time was approximately 45 min. Scores for general, numerical, verbal, and figural intelligence were calculated according to the manual. Because the BIS also included tasks loading on the operational ability creativity, we excluded those tasks to avoid artificially increasing the correlation with the creativity measurements. The final scores for numerical, verbal, and figural intelligence each consisted of four tasks; consequently, the score for general intelligence included twelve tasks. For all analyses, z-standardized scores were used.

#### 2.2.3. Mathematical Competence

Mathematical competence was measured with the mathematics test for selection of personnel (M-PA). This test was originally constructed to assess mathematical abilities for job applications and was developed to measure mathematical competencies of people with at least a lower secondary education degree (Jasper and Wagener 2013). We administered the official short form, which consists of 12 different tasks with a total of 31 items covering diverse mathematical operations including division tasks, potencies, logarithms, roots, fractions, and multiple-choice questions regarding the mathematical knowledge of a person. Problems were either multiple-choice or open answer format. The short version has a good internal consistency (Cronbachs α = 0.89) and correlates very highly (*r* = 0.93) with the long version (Jasper and Wagener 2013). Following the instructions, participants had 15 min to work on the problems. Questions were scored according to the manual as correct or incorrect. A total score (range: 0 to 31 points) was computed, and for further analyses, the z-standardized score was used.

#### 2.2.4. Domain-General Creativity

To assess domain-general creativity, we used two verbal and two figural tasks of the Torrance Test of Creative Thinking (TTCT; Torrance 1966). Participants had to finish every task within 3 min. In the verbal tasks, subjects had to find unusual ways of using a tin can (unusual uses) and write down consequences of an unlikely situation in which a suddenly appearing fog on Earth covers everything visible (Just suppose). In the first figural task, people had to complete ten incomplete pictures by drawing (Picture completion). At the end, all pictures should tell a coherent story. In the second figural task, as many figures as possible should be drawn from empty circles. The circle had to represent the central element of what the respective picture should show (Circles). Task performance was scored according to the test manual (Torrance 1966) with respect to fluency (i.e., the number of generated ideas), flexibility (i.e., the number of different categories of ideas), and originality (i.e., the novelty/creativity of ideas). For the evaluation of originality, there were detailed instructions in the TTCT manual for assigning points to each generated idea. The internal consistency across all twelve measurements of the TTCT (fluency, flexibility, and originality of each of the four tasks) was good in this sample (α = 0.87). The values from all three scores of all four tasks were summed to a total score of general creativity. For further analyses, this score was z-standardized.

### 2.3. Procedure

Subjects performed all tests in paper-and-pencil format in group sessions of up to four participants. There were two types of tasks in this study: tasks for convergent thinking (CT; intelligence and mathematical competence) and tasks for divergent thinking (DT; mathematical creativity and general creativity). To control possible sequence effects, the sequence of the task type blocks (CT, DT) was randomized across test sessions. Furthermore, the MathCrea included the numerical and figural mathematical tasks described above. Again, to control possible sequence effects, the presentation order of the task domains was pseudo-randomized. One half of the participants completed the MathCrea tasks in the following order: Figural Generate—Numerical Generate—Figural Similarities—Numerical Similarities. The other half followed this order: Numerical Similarities—Figural Similarities—Numerical Generate—Figural Generate. All tasks were administered based on written instructions on the first page of every test. After reading the instruction, participants had to fill out each task within a given time frame. After finishing a respective test, the experimenter handed out the next test to the participants. The duration of the entire study was approximately two hours.

### 2.4. Analyses

Considering that the majority of our mathematical creativity tasks have never been used in adults, we analyzed the psychometric properties of the MathCrea and did an exploratory factor analysis to investigate the underlying structure of our measurement. A confirmatory factor analysis was done afterwards with the second order factor mathematical creativity to assess the construct validity. To assess the fit of our model, we used the Chi-Square (χ^2^) and degrees of freedom (df) ratio, the comparative fit index (CFI), the Tucker Lewis Index (TLI), and the root mean square error of approximation (RMSEA). According to Schreiber et al. (2006), we considered CFI > 0.95, TLI > 0.0.95, RMSEA < 0.08, and χ^2^/df < 3 as a good model fit. The relationships between mathematical creativity and the other constructs of intelligence, mathematical competence, and general intelligence were, in a first step, examined by performing Pearson correlations. In a second step, we conducted multiple linear regressions with mathematical creativity as the outcome variable and the sub-facets of intelligence, mathematical competence, and general creativity as predictors. The method of predictor selection was “Enter”, in which all predictors are forced into the model simultaneously. Analyses were done using IBM SPSS 26 and Jamovi.

## 3. Results

### 3.1. Psychometrics and Structure of the MathCrea

All tasks of the mathematical creativity measure displayed substantial variance and no evidence of ceiling effects (see Table A4), suggesting that the measure had a satisfactory sensitivity. The reliability of the MathCrea was evaluated by determining the internal consistency in terms of Cronbach’s alpha. This revealed a high internal consistency (α = 0.85) across all twelve measurements of the MathCrea (fluency, flexibility, and originality of each of the four tasks).

To investigate the factors structure of the mathematical creativity measure, an exploratory factor analysis (EFA) with oblique rotation was performed. Because the multivariate normality assumption was violated, we used the extraction method “principal axis”, which will, according to Costello and Osborne (2005), lead to the best results. The number of factors was decided based on a parallel analysis and the visual inspection of the Scree Plot. This revealed a four-factor solution (RMSEA = 0.13, TLI = 0.92, χ^2^ = 52.41, df = 24, χ^2^/df = 2.18). The scoring components (i.e., fluency, flexibility, and originality) of every task loaded on one latent factor each. All four factors explained 84.84% of variance in total. The correlation matrix of the factors showed that all factors were positively correlated (*r* ≥ 0.15), except for the factor Numerical Generate, which showed low correlations especially with the factor Numerical Similarities (*r* = 0.01). This suggests that it shares little common variance with the other mathematical creativity measures (see Table A2). Therefore, a second EFA with the same settings as before was conducted only using the scoring components of the three items Numerical Similarities, Figural Generate, and Figural Similarities. A three-factor solution seemed to fit the data best (RMSEA = 0.08, TLI = 0.98, χ^2^ = 17.70, df = 12, χ^2^/df = 1.48) and, again, the scoring components (i.e., fluency, flexibility, and originality) of every task loaded on one latent factor. All three factors explained 86.76% of variance in total. The correlation matrix of the factors showed that all factors were positively correlated (*r* ≥ 0.19; see Table A3). Based on the amount of explained variance, the fit indices, and a reanalysis of the internal consistency including only the measurements of the remaining three tasks (*N* = 9, α = 0.88), we decided to exclude the task Numerical Generate from further analyses. The results from the EFA provided evidence that all three items captured correlated but also reasonably distinct parts of mathematical creativity.

**Figure 3:**
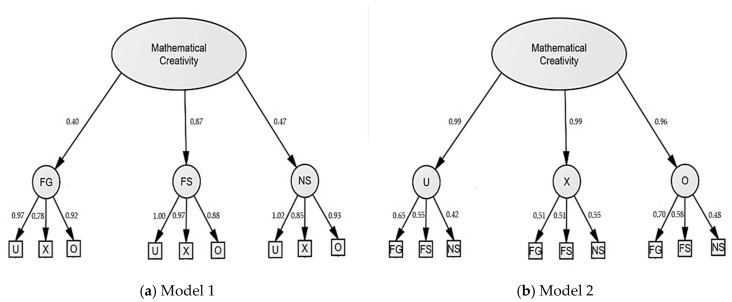
Comparison of two models: (**a**) CFA with the three tasks Figural Generate (FG), Figural Similarities (FS), and Numerical Similarities (NS) as first order factor and mathematical creativity as second order factor; (**b**) CFA with the measurements Fluency (U), Flexibility (X), and Originality (O) as first order factor and mathematical creativity as second order factor. Factor loadings in this image are standardized. Error terms as well as error covariances between the manifest variables, which account for their overlap, are not shown to increase clarity. Factor loadings in this image are standardized.

In a next step, we compared two models using CFA (depicted in Figure 3). The first model was motivated from the results of the previous EFA. The scoring components of the three tasks were used as observed variables, the three tasks Numerical Similarities, Figural Generate, and Figural Similarities constituted three first order latent variables, and the model was topped by a second order factor “mathematical creativity”. Overall, Model 1 showed a good model fit to the data (CFI = 0.990, TLI = 0.970, χ^2^ = 25.525, df = 15, χ^2^/df = 1.702, *p* = 0.043, RMSEA = 0.084 (90% CI: 0.015–0.139)). The structure of the second model was motivated from previous studies in children (Schoevers et al. 2018; Kattou et al. 2013). The scoring components of the three tasks were used as observed variables, the three measurements fluency, flexibility, and originality constituted three first order latent variables, and the model was topped by a second order factor “mathematical creativity”. Model 2 also showed a good model fit to the data (CFI = 0.996, TLI = 0.987, χ^2^ = 19.686, df = 15, χ^2^/df = 1.312, *p* = 0.043, RMSEA = 0.056 (90% CI: 0.000–0.117)). Model 2 (AIC = 97.69, BCC = 106.45) had a lower Akaike Information Criterion (AIC) and a lower Browne–Cudeck Criterion (BCC) compared to Model 1 (AIC = 103.53, BCC = 112.30), which indicated a better fit of the data. Both models, especially Model 2, suggested that the nine scores reflect an underlying latent variable of mathematical creativity, which justified the computation of a total average mathematical creativity score, which was used in subsequent analysis.

### 3.2. Relationship of Mathematical Creativity with Other Constructs

To explore the relationships between mathematical creativity and intelligence, mathematical competence, and general creativity, we used Pearson correlations (all variables were normally distributed; see Table A5). The results revealed a positive association between the MathCrea score and general intelligence, thus demonstrating that the better adults performed in the MathCrea*,* the higher they scored in the intelligence test. Within the intelligence domains, mathematical creativity showed medium sized correlations with verbal and numerical intelligence, and a slightly lower correlation with figural intelligence. The results revealed a small to medium sized positive association between the MathCrea score and mathematical competence. Further, MathCrea scores were significantly correlated with general creativity scores, indicating that the better individuals scored in the mathematical creativity measure, the better they scored in the general creativity test (see Table 1).

Due to the intercorrelation of the variables related with mathematical creativity and because we were interested in the unique contribution of each specific ability, we conducted a multiple linear regression. Mathematical creativity was defined as outcome variable, and verbal intelligence, numerical intelligence, figural intelligence, mathematical competence, and general creativity as predictors. The multiple regression showed that the additive effect of all five predictors could significantly predict mathematical creativity (*R*^2^ = 0.35, *F*(5,94) = 10.26, *p* < 001). General creativity and numerical intelligence explained a significant amount of variance in mathematical creativity beyond the other predictors, while verbal intelligence, figural intelligence, and mathematical competence did not significantly predict the mathematical creativity score beyond the other predictors (see Table 2).

### 3.3. Exploratory Analyses of Quantitative and Qualitative Aspects of Mathematical Creativity

One well-known and debated challenge in the scoring of creativity tests is a potential confound of quantity (fluency) and quality (originality) of the responses (Silvia et al. 2008). Most creativity tests (e.g., TTCT; Torrance 1966; MCT; Kattou et al. 2013) conceptualize originality as a measurement of uniqueness and assign points to every response depending on the response frequency in relation to the sample under investigation. Thus, with an increasing number of responses, the likelihood of generating a unique response also increases. Further, originality scores in most creativity measurements are summative. Therefore, those scores correlate very highly with fluency scores due to their dependence on the number of responses, which undermines the discriminant validity of originality scores (Forthmann et al. 2020; Hocevar 1979). Proposed solutions for the fluency confound are that independent raters should score originality as an alternative to infrequency (Silvia et al. 2008). Further, computing originality scores that are independent of response number such as average scores, top-scores, etc. (Benedek et al. 2013; Silvia et al. 2008), can address the fluency bias.

Here, we performed additional analyses that consider this aspect and estimated quantitative and qualitative aspects of mathematical creativity (i.e., the latter being not biased by quantitative aspects). To this end, we computed a quantitative mathematical creativity score, which is comprised of the fluency and flexibility scores of all four items (eight variables in total) and a qualitative mathematical creativity score, which includes the average originality score as measured by infrequency and the averaged rated originality of all four tasks (eight variables in total). Internal consistency for the quantity aspect was acceptable (α = 0.75) but improved to good internal consistency after removing the two scores of the Numerical Generate task (α = 0.80). Internal consistency for the quality aspect was acceptable (α = 0.74) but improved after removing the two scores of the Numerical Similarities task (α = 0.77). Considering that α usually increases with the number of items, the exclusion of these items seemed reasonable. Evidence that the quantitative and the qualitative score assessed aspects of mathematical creativity separably is provided by an only moderate correlation between those two measurements (*r* = 0.37, *p* < 0.001). Moreover, the correlation between fluency and the averaged originality score is significantly lower than the correlation between fluency and the summative originality score (see Table A7).

We used Pearson correlations to explore the relationships of quantitative and qualitative mathematical creativity with intelligence, mathematical competence, and general creativity (see Table 3). Quantitative mathematical creativity is positively correlated with all measures, the correlational strength was similar to the results found on the general creativity score. In contrast, qualitative mathematical creativity is only positively correlated with general intelligence and verbal and figural intelligence.

In a second step, we again conducted a multiple linear regression analysis with quantitative or qualitative mathematical creativity as outcome variable and verbal intelligence, numerical intelligence, figural intelligence, mathematical competence, and general creativity as predictors. The regression for quantitative mathematical creativity showed that general creativity and numerical intelligence explained a significant amount of variance in mathematical creativity (*R*^2^ = 0.35, *F*(5,94) = 10.01, *p* < 0.001). The regression for qualitative mathematical creativity, in contrast, showed that none of the predictors explained a significant amount of unique variance beyond the other predictors in mathematical creativity (*R*^2^ = 0.08, *F*(5,94) = 2.65, *p* = 0.028). The multiple regression results are depicted in Table 4.

## 4. Discussion

The aim of the current study was to develop and evaluate a mathematical creativity measure for adults (MathCrea) and to exploratorily investigate the relationship of mathematical creativity with measures of intelligence, mathematical competence, and general creativity.

### 4.1. Evaluation of the MathCrea

Overall, the MathCrea demonstrated a good reliability with a Cronbach’s α = 0.88. An EFA revealed that the tasks of the mathematical creativity measure group into three correlated factors, which were each constituted of fluency, flexibility, and originality of the respective task. The comparison of two models, one derived from the results of the EFA and one derived from a theoretical standpoint, showed that a model with the three first-order factors of fluency, flexibility, and originality, which load on a second order factor of mathematical creativity, explained the data best. The high correspondence between the theoretical driven model and the data points toward a good construct validity of the MathCrea measure.

The inter-correlations between tasks, as well as the EFA, showed that the task Numerical Generate (“Use the numbers 2, 3, 8 and the symbols +, −, (), ×, /, to create as many correct equations as possible.”) did not measure the construct to the same degree as the other three tasks; consequently, it was excluded from further analyses. One reason that could explain why this item showed weak associations was the way it was adjusted to fit an adult sample. To increase the difficulty for adults, we imposed additional restrictions that limited the solution space: (a) the same number can be used a maximum of two times in a row and (b) no operation symbols are allowed in the solution. Although similar items are frequently used in mathematical creativity research (e.g., “Use the symbols +, −, ×, ÷ and (), if needed, to write as many true equations as possible with the numbers 2, 5, 9 using them in same or different order.“, (Chauhan 1984); “Fill in the blanks by using the digits 1, 2, 3, 4, 5, 6 and the mathematical symbols +, −, ×, ÷, () in order to create an equality. Find as many equalities as possible.”, Kattou et al. 2013), the additional restrictions could have transformed the item. First, the additional restrictions seemed to have transformed the item into one that was partly ambiguous. In line with this assumption is the observation that participants did not always manage to comply with the imposed restrictions and, therefore, several solutions had to be excluded as incorrect. Thus, the number of invalid responses in this task was much higher compared to the other tasks. Second, the additional restrictions may have left less room for creative thinking and assessed rather reasoning or logical thinking. Additional evidence for this explanation comes from the result that in contrast to all other items, Numerical Generate did not correlate significantly with general creativity (see Table A7). General creativity has been shown to be an important prerequisite for creative performance in the domain of mathematics, because it facilitates the original combination of ideas and the consideration of unusual approaches (Pitta-Pantazi et al. 2018). Consequently, if the item Numerical Generate would measure mathematical creativity, it should have correlated positively with general creativity, as the other items did. 

Concerning the structure of the MathCrea, our findings are in line with results from other mathematical creativity tests constructed for children (e.g., Kattou et al. 2015; Kattou et al. 2013; Schoevers et al. 2018; Zainudin et al. 2019). These studies used CFA to confirm that the latent variable mathematical creativity underlies the observed indicators fluency, flexibility, and originality. For instance, Kattou et al. (2013) obtained the fluency, flexibility, and originality scores by adding the respective scores across five creativity items and showed that mathematical creativity is a first order latent variable. The studies by Schoevers et al. (2018); Kattou et al. (2015); as well as Zainudin et al. (2019) used mathematical creativity as second order variable, fluency, flexibility, and originality as first order latent variables, and the corresponding measurements as observed variables, and found a good model fit to the data.

Nevertheless, there is also evidence from a recent paper on mathematical creativity in children that suggests that separate questions of a mathematical creativity task each measured a unique part of mathematical creativity, and scores should better be nested within tasks. This finding is actually in line with the results from our EFA. However, it seems possible that this factor structure actually depends on the specific method of task scoring. The most obvious reason why a model suggests that fluency, flexibility, and originality of each task load onto one factor is that the high correlation between those variables was likely caused by a fluency confound. Both flexibility and originality usually increase when many answers are given, especially for summative scorings (Silvia et al. 2008; Torrance and Safter 1999). Recent research has tried to disentangle the artifactual and potential true part of the observed correlation (Forthmann et al. 2020). As an alternative approach, originality scores can be adjusted for fluency to ensure discriminant validity of scores, as has been done by this study (see Section 4.3).

### 4.2. The Association between the MathCrea and Intelligence, Mathematical Competence, and General Creativity

Mathematical creativity showed a positive relationship with intelligence, mathematical competence, and general creativity, but only general creativity as well as numerical intelligence explained the unique variance of mathematical creativity, as shown by a multiple regression analysis. The positive relationship between intelligence and mathematical creativity in adults is in line with previous research in children and adolescents (e.g., Kroesbergen and Schoevers 2017; Karwowski et al. 2020; Kattou et al. 2013; Leikin and Lev 2013). Those studies have used a variety of different measurements as well as diverse samples; therefore, this association seems to be quite generalizable. Additionally, the strength of the relationship (*r* = 0.47) is comparable to studies in children (e.g., *r* = 0.53, Kroesbergen and Schoevers 2017). An explanation for why this fundamental association exists could be that both intelligence as well as mathematical creativity heavily rely on the ability to solve problems effectively and efficiently (Plucker et al. 2015).

Multiple regression analyses revealed that only numerical intelligence but not verbal and figural intelligence significantly predicted mathematical creativity in adults. Considering that we measured creativity in the domain of mathematics using numerical items, it seems plausible that the intelligence dimension of the respective domain is the most relevant predictor. This corroborates the convergent validity of the test, whereas the non-significant association with verbal and figural intelligence after controlling for numerical intelligence offers evidence for discriminant validity. It should be noted, however, that this pattern was only observed at the level of regression analysis but not in the correlational results. Verbal intelligence (*r* = 0.36) correlated similarly strongly as numerical intelligence (*r* = 0.35) with mathematical creativity; the correlation with figural intelligence (*r* = 0.27) was significantly lower. We hypothesize that this correlation-regression pattern can be accounted for by the intercorrelation between the three sub-facets of intelligence (see Table 1). All three intelligence domains include the same operational abilities, namely processing speed, memory, and reasoning, which are domain-general processes. Processing speed and reasoning are also essential for producing creative results (Forthmann et al. 2019). Therefore, those shared processes may have driven the correlation between verbal and figural intelligence and mathematical creativity. When the shared processes of the intelligence domains are controlled for, as is done by the multiple regression analysis, the unique domain-specific components of the intelligence domains can be better dissociated, indicating that the numerical part of numerical intelligence, rather than the intelligence part, was responsible for the predictive value on mathematical creativity.

Our finding of a relationship between mathematical competence and mathematical creativity in adults is similar to previous studies in children (e.g., Sak and Maker 2006; Kattou et al. 2013; Schoevers et al. 2018; Stolte et al. 2018). Most studies in children showed correlations up to *r* = 0.63 between mathematical competence and mathematical creativity and demonstrated that mathematical competence is the strongest predictor for explaining variance in mathematical creativity (Mann 2005). However, the correlation we found was considerably smaller (*r* = 0.24), also in comparison to a sample of college students (*r* = 0.35, Chen et al. 2006), and did not significantly predict mathematical creativity scores beyond the other measures. A similar finding was recently reported by Stolte et al. (2020) with a small manifest correlation (*r* = 0.20) between mathematical ability and mathematical creativity, which became statistically insignificant when taking into account other factors in an SEM. Our finding could be partly explained by the high correlation between the sub-facets of intelligence and mathematical competence. Intelligence is highly correlated with school performance (Deary et al. 2007), and the M-PA that we used to measure mathematical competence assesses higher mathematical knowledge typically learned in secondary school. Consequently, when controlling for intelligence, the shared variance is removed, and the impact of mathematical competence becomes insignificant. Further, Schoevers et al. (2018) used a standardised mathematical competence test including not only complex mathematical topics (e.g., geometry) but also more basic processes (e.g., mental arithmetic). In their study, mathematical competence could significantly predict mathematical creativity even when intelligence was used as a covariate. Hence, the complexity of mathematical knowledge, which was assessed with the M-PA, may have been too high compared to the level of mathematical knowledge that is needed for the successful completion of the MathCrea. This leads to the assumption that the link between creativity and mathematical competence, while controlling for intelligence, becomes relevant when one uses numerical competence tasks that have a lower demand on intelligence than the M-PA did. To overcome this mismatch in future studies, mathematical competence should be assessed more broadly. For example, Kroesbergen and Schoevers (2017) demonstrated that number sense, assessed with a symbolic magnitude comparison task, a non-symbolic magnitude comparison task, and a number line task, could successfully predict mathematical creativity. Therefore, future assessments of mathematical abilities should include not only higher-order mathematical competencies, but also arithmetic skills as well as basic numerical abilities.

Our findings showed that general creativity was the most important predictor for mathematical creativity. This provides some support towards a domain-general view of creativity, suggesting that general creativity is an important prerequisite for being creative in the domain of mathematics. General creativity is thought to help individuals to combine ideas and consider alternative approaches to a situation in original ways (Pitta-Pantazi et al. 2018). While recent studies in children suggested that general creativity and mathematical competence are almost equally important for mathematical creativity (Schoevers et al. 2018; Stolte et al. 2020), our results revealed general creativity as the strongest predictor for mathematical creativity. However, the results are not completely consistent. Schoevers et al. (2018) found general creativity to be a significant predictor of mathematical creativity (*r* = 0.40), but Stolte et al. (2020) reported an insignificant relationship (*r* = 0.14). Our finding may be explained by the relatively low impact of mathematical competence. Because our participants were adults and therefore considerably older than the participants from other studies, who were children, they also had higher mathematical competences. However, the mathematical requirements of the MathCrea and of the measurement of mathematical creativity used for children were approximately equal. Consequently, in adults, mathematical competences became less important for being mathematically creative, leading to general creativity becoming relatively more important. Solely focusing on the strength of general creativity as a predictor, our results (*r* = 0.42) are comparable to those found from Schoevers and colleagues. Knowing that their study used the same items from the TTCT (Torrance 1966) as we did, this seems plausible. Stolte et al. (2020), however, only used one figural item to assess general creativity, which could have prevented them from capturing the entire concept of general creativity abilities (Cropley 2000).

### 4.3. Quantitative and Qualitative Aspects of Mathemematical Creativity

The aggregation of all creativity test variables into one creativity score is a standard procedure in mathematical creativity research. However, this procedure may obscure the role of quantity versus quality in creative performance. To overcome this shortcoming, we operationalized originality not only by infrequency, but also through creativity ratings, which are thought to be more sensitive to the creative quality of responses (Silvia et al. 2008). Furthermore, we took the fluency confound into account by computing average originality scores (evidence that the averaged originality scores avoid the fluency confound can be found in Table A7). Together, this enabled independent analyses for the quantitative as well as qualitative aspects of mathematical creativity. Quantitative and qualitative mathematical creativity were only moderately correlated, indicating that they assess aspects of the construct of mathematical creativity separably.

Quantitative mathematical creativity was operationalized with fluency and flexibility. The results for quantitative mathematical creativity were analogues to the results from the general mathematical creativity score: General creativity and numerical intelligence significantly predicted quantitative mathematical creativity scores. This finding supports the notion that a composite creativity score involving summative originality scores essentially reflects the fluency of creative production.

Qualitative mathematical creativity was operationalized with averaged infrequency scores and averaged creativity ratings. Correlation analyses revealed that qualitative aspects of mathematical creativity showed lower correlations with all factors: the correlations with verbal intelligence and figural intelligence still were significant. Numerical intelligence (*p* = 0.076), mathematical competence (*p* = 0.061), and general creativity (*p* = 0.060) scratched the significance level. Interestingly, the regression analysis showed that none of the above-mentioned variables predicted unique variance in qualitative mathematical creativity. While general creativity was the strongest unique predictor for quantitative aspects of mathematical creativity, it did neither significantly correlate nor uniquely predict qualitative mathematical creativity. This is in line with Singh (1987) as well as Walia and Walia (2017), who found that general creativity was significantly correlated with fluency and flexibility in children but not with originality. Walia and Walia (2017) argued that the absence of a significant correlation is content-based. They assume that originality in mathematical creativity depends on content knowledge (i.e., numerical intelligence, mathematical abilities), while originality in general creativity does not. Hence, no correlation between the two is expected. A student can have a high general creativity but low mathematical creativity because of the lack of the required content knowledge. Another possible explanation why general creativity did not uniquely predict qualitative mathematical creativity was the measurement of general creativity. General creativity was measured with the TTCT, which employs a summative scoring that is prone to the fluency confound, and thus reflects the quantity of creative production rather than its quality, and quantity and quality should be distinct aspects of creativity. Stolte et al. (2018) evaluated the impact of mathematical ability on fluency, flexibility, and originality separately and showed that mathematical ability had the lowest predictive value for originality compared to fluency and flexibility; yet originality was not corrected for the fluency confound. Based on the review of literature (e.g., Walia and Walia 2017), we would have expected qualitative mathematical creativity to be predicted by mathematical competence, which was not the case, although the standardized regression coefficient was marginally larger for qualitative mathematical creativity (β = 0.09) compared to quantitative mathematical creativity (β = 0.06), albeit still insignificant. 

Interestingly, the largest significant correlation with qualitative mathematical creativity was verbal intelligence, whereas numerical intelligence, but not verbal intelligence, independently predicted quantitative mathematical creativity. This finding points to an interesting conceptual dissociation supporting the discriminant validity of these two facets of mathematical creativity. Verbal intelligence and also verbal fluency are known to play an important role for the generation of creative ideas, and this relationship actually tends to be higher for predicting the originality than the fluency of ideas (Benedek et al. 2017; Silvia et al. 2013). Moreover, verbal ability was also shown to be highly relevant to the evaluation of creative ideas (Benedek et al. 2016). These relationships are partly explained by the verbal nature of most creativity tasks, which require the participant to elaborate responses in the verbal domain. We can only speculate why these findings generalize to mathematical creativity in the numerical and figural domain. First, the similarity tasks involved at least some verbal component for expressing ideas. Hence, the ability to verbalize and aptly describe connections between numbers or figures may support task performance. Second, verbal abilities may also play a role in effectively interpreting verbal task instructions. In fact, participants had some troubles attending to the rules of the Numerical Generate task, and successful performance in this task was found to be more relevant to qualitative than quantitative mathematical creativity. Ultimately, it should be noted that verbal intelligence played an important role for all measures of mathematical creativity at the manifest level, and the differences between intelligence facets may deserve more attention in future research.

### 4.4. Limitations and Future Directions

The present findings should be interpreted in light of the limitations of this study. First, it has to be kept in mind that although we speak of mathematical creativity, we actually only measured divergent thinking in the domain of mathematics. We did not measure convergent thinking, which is often considered as an essential part of creativity (Cropley 2006). Haylock (1997) proposed the process of overcoming fixations as one of the key cognitive processes in creative problem solving in mathematics, which could be measured by insight tasks in the numerical domain (Dow and Mayer 2004). Second, some of the creativity tasks do not focus on finding solutions for a given problem but rather ask what problem could be solved with this solution. For instance, in the well-established alternate uses task, participants are required to find original uses for an object. This also partly applies to some of the mathematical creativity problems (e.g., find similarities between the numbers 16 and 36—e.g., both numbers are prime numbers if one is added). It may be more realistic to present people with a more specific problem (e.g., find numbers which become prime numbers if one is added) for which they have to find the respective solution (e.g., 16, 36). While problem finding is viewed an important aspect of creativity (e.g., Chand and Runco 1993), it still raises the question how a focus on problem finding versus problem solving in the assessment of creativity affects the results. Future studies could consider the use of such more realistic mathematical creativity tasks. One example of this approach are multiple solution tasks (MST), where a complex mathematical problem has to be solved in different ways (e.g., Leikin and Lev 2013; Leikin et al. 2017; Leikin 2009). Third, the number of mathematical creativity items we used was relatively limited. Although the reliability of the MathCrea was good, a larger number of items drawing on different aspects of mathematical thinking may reveal different result patterns with regard to the relationship to other constructs. Fourth, originality was scored differently in the TTCT and in the MathCrea. We used a combined originality score (infrequency scores from 1 to 5 based on our sample plus ratings from 1 to 5 from five independent raters) in the MathCrea, while we scored originality in the TTCT according to the manual (infrequency scores from 0 to 1 or from 0 to 2, depending on the task, based on a representative norm sample). This leaves room for the question to what extent the scoring affects the construct representation. Future studies should consider this methodological issue and use a consistent scoring scheme for originality in all creativity tasks. Fifth, although we found significant predictors for the mathematical creativity scores, they could only account for 35% of the variance (respectively, only 8% for the qualitative aspect of mathematical creativity and no significant unique predictors), consequently leaving a large amount of unexplained variance. Subsequent work is needed to investigate other possible predictors for mathematical creativity. Potential candidates are executive functions (Stolte et al. 2018; Stolte et al. 2020) and number sense (Kroesbergen and Schoevers 2017). Finally, a larger sample size would have opened up the possibility for more complex statistical methods, like SEM, as well as increased the power of our analyses. Additionally, subsequent work including a second sample, with individuals from a more diverse population and not only students, is needed to cross-validate and prove the stability of our findings and also open up the possibility for more complex statistical methods, like SEM.

Despite these limitations, the current study provided a first exploratory look at mathematical creativity in adults and its relationship to different facets of intelligence (especially numerical intelligence), mathematical competence, and general creativity. While intelligence and general creativity seem to be important predictors for mathematical creativity in adults, comparable to the results found in children, mathematical competence seems to be not as important for mathematical creativity in adults as in children. Further analyses separating quantitative and qualitative aspects of mathematical creativity revealed dissimilar relationships to intelligence components and general creativity, offering more differentiated insights into the underlying associations and suggesting that they can be moderated by the type of assessment approach.

## Figures and Tables

**Table 1:** Pearson correlation coefficients between mathematical creativity, general intelligence and its sub-facets (verbal, numerical, and figural), mathematical competence, and general creativity.

	Mathematical Creativity	General Intelligence	Verbal Intelligence	Numerical Intelligence	Figural Intelligence	Math Competence	General Creativity
Mathematical Creativity	1						
					
General intelligence	0.43 **	1					
				
Verbal intelligence	0.36 **	0.73 **	1				
			
Numerical intelligence	0.35 **	0.75 **	0.40 **	1			
		
Figural intelligence	0.27 *	0.75 **	0.35 **	0.26 *	1		
	
Math competence	0.24 *	0.47 **	0.32 **	0.43 **	0.30 **	1	

General creativity	0.42 **	0.07	0.27 *	−0.06	0.01	−0.04	1

Significance levels: * indicates *p* < 0.050; ** indicates *p* < 0.002 (Bonferroni corrected threshold 0.050/21).

**Table 2:** Multiple regression model for mathematical creativity predicted from verbal intelligence, numerical intelligence, figural intelligence, math competence, and general creativity.

	Predictor	β	*t*	*p*
Mathematical creativity	Verbal intelligence	0.06	0.56	0.577
	**Numerical intelligence**	**0.28**	**2.84**	**0.006**
	Figural intelligence	0.16	1.75	0.083
	Math competence	0.07	0.72	0.475
	**General creativity**	**0.42**	**4.79**	**<0.001**

**Table 3:** Pearson correlations between quantitative mathematical creativity and qualitative mathematical creativity and general intelligence and its sub-facets (verbal, numerical, and figural), mathematical competence, and general creativity.

	General Intelligence	Verbal Intelligence	Numerical Intelligence	Figural Intelligence	Math Competence	General Creativity
Quantitative mathematical creativity	0.42 **	0.37 **	0.33 **	0.26 *	0.22 *	0.43 **
Qualitative mathematical creativity	0.30 **	0.27 **	0.18	0.23 *	0.19	0.19

Significance levels: * indicates *p* < 0.050; ** indicates *p* < 0.008 (Bonferroni corrected threshold 0.050/6).

**Table 4:** Multiple regression model for quantitative/quantitative mathematical creativity predicted from verbal intelligence, numerical intelligence, figural intelligence, math competence, and general creativity.

	Predictor	β	*t*	*p*
Quantitative mathematical creativity	Verbal intelligence	0.08	0.83	0.411
	**Numerical intelligence**	**0.26**	**2.59**	**0.011**
	Figural intelligence	0.15	1.60	0.113
	Math competence	0.06	0.64	0.523
	**General creativity**	**0.42**	**4.78**	**<0.001**
Qualitative mathematical creativity	Verbal intelligence	0.12	1.02	0.309
	Numerical intelligence	0.07	0.59	0.559
	Figural intelligence	0.14	1.34	0.182
	Math competence	0.09	0.77	0.442
	General creativity	0.16	1.59	0.116

## Data Availability

The data presented in this study is available on request from the corresponding author.

## Appendix A

Flexibility was operationalized as the number of categorically different responses. Examples for the different categories and for answers within each category can be found in Table A1.

**Table A1:** Example categories and answers for flexibility for the four MathCrea tasks.

Category	Example Answers
*Numerical Generate*	
4 different components (symbols + numbers) within an equation	2 + 3 + 3 = 8 2 × 8/2 = 8
8 different components (symbols + numbers) within an equation	(((8 + 3) × 2 − 8)/2 − 3)/2 = 2 (3 + 2) × 8/(8 − 3) = 8
*Numerical Similarities*	
Above/below X	Below 50 Above 10
Divisible by X	Completely divisible by 4 Not completely divisible by 3
*Figural Generate*	
Triangles	
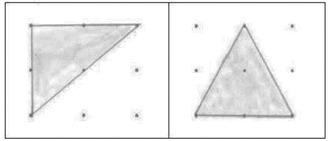
Combination of different shapes	
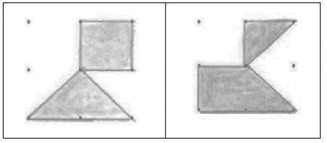
*Figural Similarities*	
Three dimensional	are three dimensional objects: A, B, C, E, F, G, H hold volume: A, B, C, E, F, G, H;
Surface area	No round surfaces: A, B, C, E, G No surface parallel areas: B, D, G

The correlation between the originality scoring based on infrequency and on ratings was moderate (Numerical Generate: *r*(98) = 0.35, *p* < 0.001; Numerical Similarities: *r*(98) = 0.29, *p* = 0.002; Figural Generate: *r*(98) = 0.41, *p* < 0.001; Figural Similarities: *r*(98) = 0.47, *p* < 0.001). Scatter plots are depicted in Figure A1.

The correlation matrix of the factors showed that the factor/task Numerical Generate correlated the lowest with the other factors (see Table A2).

**Table A2:** Correlation matrix of the four factors found with the initial EFA.

Correlation Matrix	*Figural* *Similarities*	*Numerical* *Similarities*	*Figural* *Generate*	*Numerical* *Generate*
*Figural Similarities*	1	0.40	0.32	0.20
*Numerical Similarities*		1	0.15	0.01
*Figural Generate*			1	0.25
*Numerical Generate*				1

**Table A3:** Correlation matrix of the three factors found with the second EFA.

Correlation Matrix	*Figural* *Similarities*	*Numerical* *Similarities*	*Figural* *Generate*
*Figural Similarities*	1	0.43	0.35
*Numerical Similarities*		1	0.19
*Figural Generate*			1

All tasks of the mathematical creativity measure displayed substantial variance and no evidence of ceiling effects (see Table A4), suggesting that the measure had a satisfactory sensitivity.

**Table A4:** Descriptive unstandardized statistics for the four mathematical creativity tasks.

	*Figural Similarities*	*Numerical Similarities*	*Figural Generate*	*Numerical Generate*
*M*	4.77	7.31	9.99	4.97
*SD*	2.32	3.57	5.89	2.71
*Min; max*	1.03; 12.50	2.10; 21.70	1.00; 30.80	1.03; 15.13
*Skewness*	0.83	1.19	0.62	0.91
*SE*	0.25	0.24	0.25	0.25
*Kurtosis*	1.08	1.79	0.51	1.32
*SE*	0.50	0.48	0.49	0.50

To explore the relationships between mathematical creativity and intelligence, mathematical competence and general creativity, we used Pearson correlations, since all variables were normally distributed (see Table A5).

**Table A5:** Descriptive statistics for the standardized variables mathematical creativity, general intelligence and its subfacets (verbal, numerical, and figural), mathematical competence, and general creativity. Checking for normality using the Kolmogorov–Smirnov test.

	Mathematical Creativity	General Intelligence	Verbal Intelligence	Numerical Intelligence	Figural Intelligence	Math Competence	General Creativity
*M*	−0.01	0.00	0.00	0.00	0.00	0.00	0.00
*SD*	0.73	1.00	1.00	1.00	1.000	1.00	0.66
*Skewness*	0.46	0.10	−0.50	0.19	0.31	0.07	0.28
*SE*	0.24	0.24	0.24	0.24	0.24	0.24	0.24
*Kurtosis*	−0.13	−0.52	0.33	0.32	0.64	−0.45	−0.12
*SE*	0.48	0.48	0.48	0.48	0.48	0.48	0.48
*K-S test p*	0.057	0.200	0.200	0.200	0.200	0.137	0.200

**Table A6:** Descriptive statistics for the unstandardized variables mathematical creativity, general intelligence and its subfacets (verbal, numerical, and figural), mathematical competence, and general creativity. Checking for normality using the Kolmogorov–Smirnov test.

	Mathematical Creativity	General Intelligence	Verbal Intelligence	Numerical Intelligence	Figural Intelligence	Math Competence	General Creativity
*M*	7.33	185.45	47.83	53.67	83.95	18.52	6.93
*SD*	2.98	27.34	9.66	14.90	12.44	6.37	2.16
*Min; max*	1.57; 17.32	124; 246	17; 66	21; 97	55; 126	3; 31	2.33; 13.33
*Skewness*	0.68	0.04	−0.61	0.34	0.26	0.07	0.22
*SE*	0.24	0.24	0.24	0.24	0.24	0.24	0.24
*Kurtosis*	0.58	−0.43	0.69	0.15	0.57	−0.45	−0.20
*SE*	0.48	0.48	0.48	0.48	0.48	0.48	0.48
*K-S test p*	0.090	0.200	0.200	0.140	0.200	0.137	0.200

**Table A7:** Pearson correlations for all measurements.

Variables	1	2	3	4	5	6	7	8	9	10	11	12	13	14	15	16	17	18	19	20	21	22
1 NG-U	1																					
2 NG-X	0.723	1																				
3 NG-O	0.881	0.711	1																			
4 NG-Oa	−0.137	−0.029	0.256	1																		
5 NS-U	−0.054	0.016	0.009	0.179	1																	
6 NS-X	−0.019	0.130	0.038	0.139	0.866	1																
7 NS-O	−0.013	0.015	0.052	0.202	0.944	0.788	1															
8 NS-Oa	0.146	0.056	0.166	0.093	0.378	0.281	0.634	1														
9 FG-U	0.134	0.163	0.212	0.110	0.213	0.338	0.227	0.148	1													
10 FG-X	0.077	0.028	0.190	0.163	0.121	0.244	0.132	0.094	0.770	1												
11 FG-O	0.198	0.266	0.326	0.197	0.216	0.356	0.260	0.209	0.906	0.738	1											
12 FG-Oa	0.192	0.263	0.340	0.291	0.063	0.181	0.112	0.161	0.467	0.563	0.727	1										
13 FS-U	0.154	0.146	0.216	0.119	0.437	0.383	0.469	0.362	0.350	0.282	0.351	0.151	1									
14 FS-X	0.169	0.156	0.226	0.099	0.442	0.392	0.468	0.347	0.307	0.259	0.318	0.121	0.975	1								
15 FS-O	0.094	0.108	0.225	0.230	0.408	0.378	0.454	0.367	0.362	0.316	0.408	0.278	0.892	0.861	1							
16 FS-Oa	−0.057	0.018	0.126	0.293	0.117	0.127	0.160	0.173	0.194	0.246	0.320	0.452	0.190	0.167	0.565	1						
17 gIQ	0.263	0.240	0.331	0.076	0.289	0.347	0.306	0.240	0.345	0.362	0.379	0.338	0.331	0.327	0.357	0.199	1					
18 vIQ	0.107	0.080	0.175	0.133	0.160	0.250	0.136	0.087	0.320	0.342	0.294	0.237	0.264	0.281	0.287	0.159	0.728	1				
19 nIQ	0.266	0.215	0.285	0.013	0.351	0.353	0.384	0.277	0.286	0.254	0.301	0.228	0.196	0.190	0.214	0.121	0.773	0.369	1			
20 fIQ	0.175	0.208	0.248	0.046	0.092	0.146	0.106	0.129	0.166	0.227	0.245	0.286	0.287	0.273	0.306	0.166	0.706	0.381	0.215	1		
21 m. Comp.	0.208	0.218	0.262	0.044	0.177	0.242	0.199	0.146	0.145	0.184	0.282	0.353	0.083	0.081	0.064	0.014	0.448	0.266	0.401	0.299	1	
22 gC	0.075	0.144	0.057	0.050	0.355	0.296	0.293	0.022	0.283	0.186	0.194	0.042	0.375	0.346	0.362	0.192	0.129	0.325	−0.004	0.037	−0.043	1

NG = Numerical Generate, NS = Numerical Similarities, FG = Figural Generate, FS = Figural Similarities, U = Fluency, X = Flexibility, O = Originality, Oa = Originality averaged, gIQ = general intelligence, vIQ = verbal intelligence, nIQ = numerical intelligence, fIQ = figural intelligence, m. Comp. = mathematical competence, gC = general creativity; *p* < 0.05 for *r* ≥ 0.19, and *p* < 0.01 for *r* ≥ 0.25 given that *N* = 100.
